# Plasma metagenomics reveals regional variations of emerging and re-emerging pathogens in Chinese blood donors with an emphasis on human parvovirus B19

**DOI:** 10.1016/j.onehlt.2023.100602

**Published:** 2023-07-13

**Authors:** Zhao Mengyi, Li Yuhui, Gao Zhan, Liu Anqing, Li Yujia, Li Shilin, Gao Lei, Lan Yue, Huang Mei, Wan Jianhua, He Weilan, Mao Wei, Cai Jie, Zhou Jingyu, Yin Yijing, Guo Yanli, Zhong Qiulei, Huang Yang, Chen Limin, Fan Zhenxin, He Miao

**Affiliations:** a Institute of Blood Transfusion, Chinese Academy of Medical Sciences, Chengdu, China; b Sichuan Blood Safety and Blood Substitute International Science and Technology Cooperation Base, Chengdu, China; c Shaanxi Blood Center, Institute of Xi'an Blood Bank, Xi'an, China; d College of Life Sciences, Sichuan University, Chengdu, China; e Mianyang Blood Center, Mianyang, China; f Urumqi Blood Center, Urumqi, China; g Guangxi Blood Center, Liuzhou, China; h Chongqing Blood Center, Chongqing, China; i Nanjing Blood Center, Nanjing, China; j Jiangsu Blood Center, Jiangsu Institute of Medical Biological Products, Nanjing, China; k Dehong Blood Center, Dehong, China; l Mudanjiang Blood Center, Mudanjiang, China

**Keywords:** Blood donor, Metagenomic next generation sequencing, Emerging and re-emerging infectious pathogens, Human parvovirus B19, Blood safety

## Abstract

At present, many infectious pathogens, especially emerging/re-emerging pathogens, exist in the blood of voluntary blood donors and may be transmitted through blood transfusions. However, most of Chinese blood centers only routinely screen for HBV, HCV, HIV, and syphilis. We employed metagenomic next-generation sequencing (mNGS) to investigate the microbiome in healthy voluntary blood donors to help assess blood safety in China by identifying infectious pathogens presented in donations that could lead to transfusion-acquired infections. We collected 10,720 plasma samples from voluntary blood donors from seven blood centers in different cities during 2012–2018 in China. A total of 562 GB of clean data was obtained. By analyzing the sequencing data, it was found that the most commonly identified bacteria found in the healthy blood were *Serratia spp.* (5.0176%), *Pseudomonas spp.* (0.6637%), and *Burkholderia spp.* (0.5544%). The principal eukaryote were *Leishmania spp* (1.3723%), *Toxoplasma gondii* (0.6352%), and *Candida dubliniensis* (0.1848%). Among viruses, Human Parvovirus B19 (B19V) accounts for the highest proportion (0.1490%), followed by Torque teno midi virus (0.0032%) and Torque teno virus (0.0015%). Since that B19V is a non-negligible threat to blood safety, we evaluated the positive samples for B19V tested by mNGS using quantitative polymerase chain reaction, Sanger sequencing, and phylogenetic analysis to achieve a better understanding of B19V in Chinese blood donors. Subsequently, 9 (0.07%) donations were positive for B19V DNA. The quantitative DNA levels ranged from 5.58 × 10^2^ to 7.24 × 10^4^ IU/ml. The phylogenic analyses showed that prevalent genotypes belonged to the B19-1A subtype, which disclosed previously unknown regional variability in the B19V positivity rate. The investigation revealed that many microbes dwell in the blood of healthy donors, including some pathogens that may be dormant in the blood and only cause disease under specific conditions. Thus, investigating the range and nature of potential pathogens in the qualified donations provided a framework for targeted interventions to help prevent emerging and re-emerging infectious diseases.

## Highlights

• A large amount of data was obtained, resulting in a total of 562GB of clean data.
• Microorganisms with potential risks to the blood have been discovered.
• A molecular statistical analysis was conducted on B19V, which poses a threat to blood safety.
• Beneficial for targeted prevention and control of emerging infectious diseases.

## 1 Introduction

Emerging and re-emerging infectious pathogens have gradually become publicly visible and been one of the important factors threatening the public health. Since the 1980s, a series of novel diseases (such as toxic shock syndrome, Legionnaire's disease) emerged, which eventually led to the spread of human immunodeficiency virus (HIV) around the world, emerging/re-emerging pathogens have received greater attention [1,2]. Because of the characteristics of high infectivity, wide dissemination and various modes of transmission, emerging/re-emerging pathogens cannot be prevented and treated effectively, such as severe acute respiratory syndrome coronavirus (SARS-CoV) and SARS-CoV-2, which re-shaped our world, caused high mortality and created a heavy blow to the public health system [[3], [4], [5], [6]]. Furthermore, emerging/re-emerging pathogens are present in healthy voluntary blood donors, and can be transmitted through blood transfusions, such as West Nile virus (WNV), Dengue virus (DENV), *Leishmania spp*, *Babesia spp*, *etc.* [[7], [8], [9], [10]]. Currently, the blood screening strategy for voluntary blood donations in China generally contains four pathogens, including HIV-1/2, hepatitis B virus (HBV), hepatitis C virus (HCV), and syphilis [11], respectively. Emerging/re-emerging pathogens are normally not included in routine screening, which might pose an increasingly uncertain potential threats to the blood safety [7]. Besides, unfortunately, Chinese blood centers as well as the related blood utilization hospitals lacks basic research on emerging/re-emerging pathogens. Based on the above situation, there is an urgent need for new methods to investigate the microbiome in healthy voluntary blood donors to help assess blood safety in China by identifying infectious pathogens presented in donations that could lead to transfusion-acquired infections.

Traditional blood detection methods typically identify pathogens one by one, which is time-consuming and costly [12,13]. Specifically, culture methods rely on the growth of live microorganisms in the culture for identification, taking at least 48 h for common pathogens and longer for more picky ones. The low detection rate makes it difficult for most patients to achieve accurate detection. Other traditional technologies, such as serological testing and nucleic acid testing (NAT), can only detect a small number of known pathogens. [14]. Although syndromic multiplex polymerase chain reaction (mPCR), 16S ribosomal DNA (16S rDNA) sequencing, matrix-assisted laser desorption ionization time-of-flight mass spectrometry (MALDI-TOF MS) and other new technologies have shortened the detection time, the etiology of up to 60% of infectious diseases in clinical is still unknown [[15], [16], [17]]. Therefore, the above detection methods are not suitable for emerging/re-emerging pathogens.

Metagenomic next-generation sequencing (mNGS) can efficiently detect various pathogens in one clinical sample without prior separation, culturing, and purifying of microorganisms. Therefore, it has become an ideal tool to identify unrevealed pathogens [14,18] in qualified blood donations. For example, some researchers reported that Aneloviridae, Human papillomavirus, and Herpesoviridae were found in red blood cells and plasma of healthy individuals [19,20]. Another study found thirteen bacteria, two parasites, and six nonpathogenic viruses in healthy human plasma [21]. In this study, by using mNGS, we examined the microbiome of donation samples from voluntary blood donors in seven regions of China, evaluated transfusion-transmitted risks of potential pathogens and investigated the prevalence of some important pathogens which potentially threatened the blood safety in the specific area, supplementing the data of emerging/re-emerging pathogens on blood safety in China.

## 2 Materials and methods

### 2.1 Sample collection and nucleic acid preparation

**Fig. 1:**
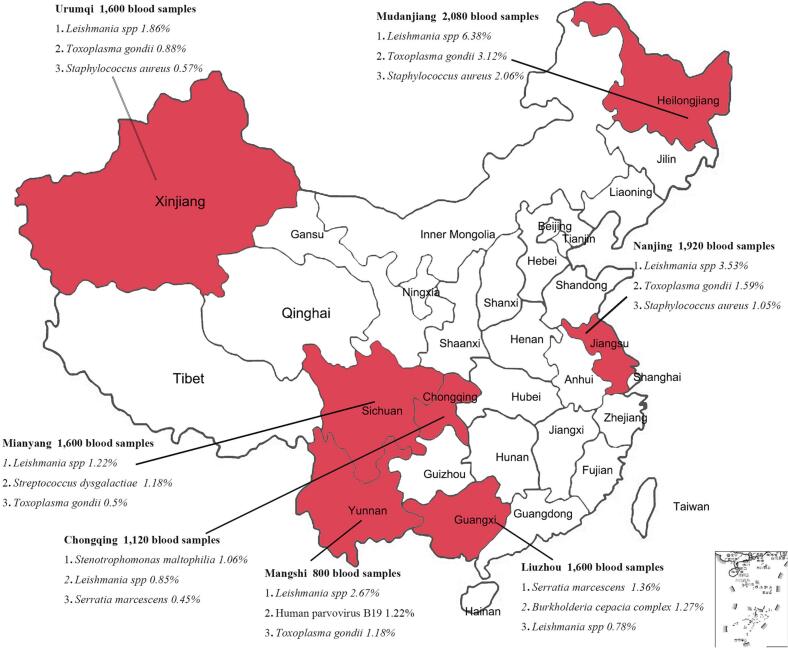
Sampling map of voluntary blood donors in seven regions of China and the distribution of potential pathogens of top three abundances in pooled plasma of blood.

From January 1, 2012, to December 31, 2018, a total of 10,720 blood samples of 10 ml each were randomly selected from voluntary blood donors in 7 regions (the distribution of sampling location and a corresponding number of samples are shown in the Fig. 1). The blood samples taken from various places were mixed in units of 160 (each 100 μl) for ultracentrifugation (32,000 rpm, 120 min, maximum centrifugal radius of 91.9 mm). Afterward, we rinsed and resuspended the precipitate with 500 μl PBS.

The pooled suspensions were subjected to extraction of total DNA using QIAamp® DNA Blood mini Kit (QIAGEN Cat. NO.160019269, Frankfurt, Germany), DNA concentration was measured by Equalbit® 1 × dsDNA HS Assay Kit (Vazyme Cat. NO. 7E302K9, Nanjing, China).

### 2.2 Library construction and metagenome sequencing

The metagenomic library was constructed using KAPA HyperPlus Kit (KAPA Cat. NO. 0000097583, Boston, USA) with dual-indexed Adapters (KAPA Cat. NO. 0000093370, Boston, USA), the DNA was fragmented to 250 bp approximately by the enzyme at 37 °C for 20 min, after end repair and A-tailing, adapter ligation, post-ligation cleanup, library amplification, and post-amplification cleanup, the library was constructed.

Agilent 2100 Bioanalyzer (Agilent Technologies, Beijing, China) was used for library quality control, and qualified DNA library was sent to the Novogene company to sequence in HiSeq 4500 (Illumina, Beijing, China), and all raw data were trimmed by Trimmomatic (version 0.39) [22] to remove adapter sequence, low-quality reads, and duplicate reads.

### 2.3 Metagenomic sequencing data analysis

After removing the human potential sequences by KneadData (version0.10.0, https://github.com/biobakery/biobakery/wiki/kneaddata) using the reference genome of humans (assembly GRCh38.p13), the taxonomic labels of metagenomic sequences were assigned using kraken2 (version 2.0.7) [23] and the microbe reads were surfaced. The criteria for positive mNGS results for bacteria, viruses, and fungi are reads >20, 1, and 1, respectively. Based on the Krona algorithm, homology comparison is performed with reference sequences of bacteria, fungi, parasites, and viruses. The unclassified reads were aligned to the NCBI database using BLASTn. The best alignment hits were used to classify the reads. Subsequently, heatmaps were generated for non-scaled, non-normalized titer data using a Pearson distance function with average linkage clustering using the program Heml (version 1.0).

### 2.4 B19V DNA extraction and nucleic acid amplification

Aliquots of plasma (20μl) from 10 blood donations were pooled, then nucleic acid extraction was conducted by Magnetic Viral DNA/RNA Kit (Tiangen, Beijing, China). Screening of samples for B19V DNA was performed by 2 × SG Fast qPCR Master Mix (Sangon Biotech, Shanghai, China) on fluorescence ration PCR instrument (CFX96, BIO-RAD, California, USA), and corresponding primers are located in conserved regions. The sequence forward primer is 5’-TGCAGATGCCCTCCACCCA-3′ and the sequence of reverse primer is 5’-GCTGCTTTCACTGAGTTCTTC-3′. Additionally, 3 μl of extracted DNA from pooled plasma was added to 17 μl qPCR mixture (10 μl 2 × SG Fast qPCR Master Mix, 0.4 μl of each diluted primer, 2 μl DNF Buffer) for amplification of B19V DNA, with the following reaction variables: pre-denaturation at 95 °C for 3 min, followed by 40 cycles of 3 s of denaturation (95 °C), 20 s of annealing (60 °C), and 30 s of extension (72 °C), with a detection of melting curves at 95 °C for 10 s, 60 °C for 15 s, and 95 °C for 30 min. This study used the National Standard for Human Parvovirus B19 Nucleic Acids Detection Kit (National institutes for food and drug control, Cat. NO. 370023–201,601, Beijing, China) as quantitation standards. Six quantitative standards (1 × 10^8^, 1 × 10^7^, 1 × 10^6^, 1 × 10^5^, 1 × 10^4^, 1 × 10^3^ IU/ml) were included in each PCR procedure. To avoid the fluorescence of primer dimer under high cycle number in samples with few or no target sequence, it is necessary to optimize the Tm value. The interpretation results follow the following principles: if the Ct value of the lowest concentration standard is >40, the experiment should be repeated. If the Ct value of the lowest concentration standard is <40, the standard curve R^2^ is within the range of 0.99–1, and the absolute deviation does not exceed 0.5 logarithmic orders of magnitude, then the experimental results are valid. Under normal negative control conditions, if the Ct value of the mixed sample is <40 and the Tm value of the fusion curve is between 80 and 84 °C, the mixed sample is considered positive and needs to be split and a single sample is taken for verification. Mixed samples with a Ct value >40 or a Tm value on the melting curve that is not between 80 and 84 °C are judged negative.

### 2.5 Confirmation test and phylogenetic analysis

B19V DNA positive samples were confirmed by nested PCR, using primers located in the NS1 gene: First round of primers (Forward: 5’-CACTATGAAAACTGGGCAATAAAC-3′, Reverse: 5’-AATGATTCTCCTGAACTGGTCC-3′), the second round of primers (Forward: 5’-ATAAACTACACTTTTGATTTCCCTG-3′, Reverse: 5’-TCTCCTGAACTGGTCCCG-3′), and then Golden Star T6 Super PCR Mix (QINGKE, Beijing, China) was used for nested PCR. Refer to the article for the reaction conditions [24]. The PCR products were sent to the Sangon Biotech (Shanghai, China) for sequencing. Mega-X (version 10.0.5) was used to construct the phylogenetic evolution tree. The Genbank accession numbers of the reference sequence are shown in the S1 Table, and No. of bootstrap replications is set to 1000, and the model/method is set to Kimura 2-parameter.

### 2.6 Statistical analysis

The software IBM SPSS Statistics 23.0 was used for statistical analysis. The chi-square test was applied to calculate the correlation between the demographic characteristics, and the *P* value <0.05 was used as the cutoff level for significance.

## 3 Result

### 3.1 Results of high through put sequencing

A total of 562 GB of clean data was obtained. The datasets presented in this study can be found in online repositories. The names of the repository/repositories and accession number(s) can be found below: https://bigd.big.ac.cn/gsa/browse/CRA006191 and the assigned accession of the submission is: CRA006191. The output of the total sequencing data is shown in S2 Table. Q20 is >95%, and Q30 is >89%. The amount of microbial genome sequence data is shown in S1 Figure.

### 3.2 Analysis of the composition of microbiome in pooled plasma in various regions

After classifying the final reads, the microorganisms were profiled to investigate the species composition of the microbiome from voluntary donors, as shown in Table 1. It shows that reads of bacteria are found to be the highest, followed by eukaryote, and finally viruses.

**Table 1:** Analysis of the microbial composition of blood samples from blood donors in seven regions of China.

Blood Centers	Reads(%)						
	Chongqing	Mianyang	Mudanjiang	Mangshi	Nanjing	Liuzhou	Urumqi
Bacteria	2,268,586 (93.07)	1,560,567 (86.35)	234,067 (51.29)	253,733 (77.03)	584,243 (72.95)	3,381,517 (92.77)	1,081,568 (84.26)
Fungi and parasites	158,288 (6.50)	212,193 (11.74)	215,003 (47.12)	67,157 (20.39)	211,083 (26.36)	236,580(6.49)	194,484 (15.15)
Virus	10,593(0.43)	34,500 (1.91)	7257 (1.59)	8492 (2.58)	5565 (0.69)	27,011 (0.74)	7528 (0.59)

### 3.3 Main microorganisms in donations in various regions

As shown in S2–4 Figure, the heat map showed that the main bacteria were *Serratia spp.*, *Burkholderia spp.*, *Streptococcus spp.*, and *Pseudomonas spp.*; and principal eukaryote were *Aspergillus oryzae*, *Leishmania spp*, and *Toxoplasma gondii (T. gondii)*. In addition, B19V accounts for a larger proportion among viruses.

### 3.4 Analysis of potentially harmful microbes in blood samples in various regions

The results of bioinformatics analysis revealed some pathogens that may have potential threats to blood safety (Table 2), which requires attention. Finally, Fig. 1 shows the geographic distribution of each region in this study and reveals the top three potential pathogens in the corresponding abundance of each region.

**Table 2:** Microbes with potential hazards and their proportions in the microbiome in mixed blood samples of voluntary blood donors in some areas of China.

Classification	Total microbe reads	The percentage in microbiome						
		Mudanjiang	Nanjing	Liuzhou	Chongqing	Mangshi	Mianyang	Urumqi
Bacteria								
*Serratia marcescens*	70,135	0.02	0.01	1.36	0.45	0.03	0.02	0.42
*Burkholderia cepacia complex*	65,845	<0.01	0.01	1.27	0.45	<0.01	0.01	0.41
*Staphylococcus aureus*	44,096	2.06	1.05	0.08	0.18	0.70	0.31	0.57
*Streptococcus dysgalactiae*	27,987	<0.01	<0.01	<0.01	<0.01	<0.01	1.18	<0.01
*Stenotrophomonas maltophilia*	33,331	0.01	0.19	0.04	1.06	0.12	0.14	0.02
*Pseudomonas aeruginos*	17,659	0.04	0.17	0.22	0.11	0.30	0.11	0.07
*Morganella morganii*	1738	<0.01	<0.01	0.01	0.03	<0.01	<0.01	0.03
Fungi and parasites								
*Candida dubliniensis*	21,833	0.70	0.40	0.12	0.11	0.33	0.14	0.22
*Candida albicans*	10,904	0.32	0.18	0.06	0.05	0.15	0.08	0.13
*Leishmania spp*	180,364	6.38	3.53	0.78	0.85	2.67	1.22	1.86
*Toxoplasma gondii*	75,044	3.12	1.59	0.27	0.30	1.18	0.50	0.88
*Trypanosoma brucei*	26,332	1.36	0.63	0.06	0.07	0.46	0.19	0.29
Viruses								
Human parvovirus B19	17,607	0.69	0.02	0.11	<0.01	1.22	0.17	<0.01
Torque teno midi virus	675	<0.01	<0.01	<0.01	<0.01	<0.01	<0.01	<0.01
Torque teno virus	207	<0.01	<0.01	<0.01	<0.01	<0.01	<0.01	<0.01

### 3.5 Molecular Epidemiology of B19V

After metagenomic investigation and subsequent analysis of the plasma samples of voluntary blood donors collected, it was found that 19 pooled plasma (3040 blood samples of voluntary blood donors) contained fragments of B19V genes, these plasma samples were drawn from six Chinese blood centers of Liuzhou (800), Mangshi (640), Mudanjiang (640), Mianyang (480), Nanjing (320) and Urumqi (160).

Initial screening was conducted by qPCR assay in pooled samples, and then positive samples were tested again and quantitated by the single-virus qPCR assay. Finally, nested PCR was used to confirm positive samples.

The standard plasmid containing the entire genome of B19V was used as a template and conducted for amplification at a range of 1 × 10^3^–1 × 10^8^ IU/ml, and a linear standard curve was generated (S5 Figure). As few as 1 × 10^2^ IU could be distinguished from background levels (S6 Figure). The melting curve (S7 Figure) showed that this in-house qPCR amplified a single principal product with a Tm range of 82–84 °C.

qPCR was used to detect B19V DNA in voluntary blood donors, and 58 specimens were originally found to be positive for B19V, and twelve specimens were excluded as false positives for the incorrect melting curve. After confirmation by nested PCR, agarose gel electrophoresis (S8 Figure), and sequencing in the NS1 gene, only 9 (0.07%) cases were identified as the infected person of B19V, the quantitative DNA levels ranged from 5.58 × 10^2^ to 7.24 × 10^4^ IU/ml. The demographics of B19V positive donors and statistics on the prevalence of B19V DNA are shown in Table 3. According to donation information, a demographic analysis was performed (S9 Figure), and it was found that there was no significant difference (*P* < 0.05) among demographic characteristics.

**Table 3:** Demographics and nucleic acid-positive prevalence information of human parvovirus B19-positive blood donors among voluntary blood donors in 7 regions.

Blood Centers	B19V DNA prevalence(%)	Donor	Sex	Age	Nationality	Education level	Profession	Donation frequency	Marital status	Viral load(IU/ml)
Mianyang	0.06	1	Female	50	Han	Junior high school	Farmer	First	Married	<10^3^
Mangshi	0.50	1	Male	24	Dai	Primary school	Other	First	Unknown	<10^3^
Mangshi		2	Female	33	Han	Junior high school	Farmer	Repeat	Unknown	2.48 × 10^3^
Mangshi		3	Male	49	Dai	Junior high school	Other	Repeat	Unknown	7.24 × 10^4^
Mangshi		4	Male	19	Han	Technical secondary school	Factory worker	First	Unknown	3.83 × 10^4^
Nanjing	0.21	1	Female	23	Han	University	office worker	First	Single	3.32 × 10^4^
Nanjing		2	Female	20	Han	University	Student	Repeat	Single	1.55 × 10^4^
Nanjing		3	Male	21	Han	University	Student	First	Single	4.85 × 10^4^
Nanjing		4	Female	19	Han	University	Student	Repeat	Single	6.06 × 10^4^

The 218 bp fragment amplified by nested PCR in the NS1 gene was sent to the company for sequencing, and then the sequences were aligned and used to construct a Neighbor-Joining tree. According to the Phylogenetic tree, all B19V detected in this study belong to the B19-1A subtype (S10 Figure).

## 4 Discussion

Emerging/re-emerging pathogens could pose a great threat to the safety of blood transfusions, so some European and American countries have carried out blood tests for some of them in high-risk areas and epidemic seasons [25]. mNGS has distinctive advantages in microbial research, which can be used for the identification of all pathogens and investigation of their genomic characterization [14]. Therefore, there have been many studies that used mNGS to investigate transfusion related emerging/re-emerging pathogens, which revealed some emerging/re-emerging pathogens existed in healthy voluntary blood donors, such as *Coxiella burnetii*, *S.suis*, *Orientia tsutsutsugamushi*, Herpesviruses [20,21,26], Torque teno virus, *T. gondii*, *Staphylococcus.spp* and *Pseudomonas.spp* [[27], [28], [29]].

By applying mNGS to this study, we found that there were opportunistic pathogenic bacteria in the plasma of healthy voluntary blood donors, including *S. marcescens*, *B. cepacia*, *S. aureus*, and *S. dysgalactiae*, *etc.*, which could affect the health of individuals with low immunity. *S. marcescens* can cause sepsis when human immune function is reduced [30]. *M. morganii* and *P. aeruginosa* can induce bacteremia in patients with low immunity under certain conditions [31,32]. Our research also found the principal eukaryote were *Candida dubliniensis*, *Leishmania spp*, and *T. gondii*, causing transfusion transmitted diseases occasionally. There have been cases of infection with *Leishmania.spp* through blood transfusions in developed countries in Europe and America [33], and *T. gondii* have been found in blood products throughout the world [34,35], both of which can cause asymptomatic infections among healthy blood donors in epidemic areas [36], fatal for individuals with weakened immune function. As for transfusion transmitted viruses, some literature suggested that Anelloviruses*,* Human pegivirus-1 (HPgV-1), and Human pegivirus-2 (HPgV-2) were commensal viruses in the human blood [37], but the herpesviruses, B19V and other viruses found in this study still posed a threat to blood transfusion safety [20,27,28]. In addition, some of the pathogens that we detected are also zoonotic pathogens, such as *Leishmania spp., T. gondii,* and *E. coli* [38]. Zoonosis is still a major threat to global health, and zoonotic pathogens that can be transmitted through blood, such as hepatitis E virus [39], *Brucella spp.* [40], and *S. suis* [26], also threaten the blood safety. Monitoring zoonotic pathogens transmitted through blood may help to better track the origin of pathogens, improve the understanding of the disease and its related risks, and thus effectively respond to health threats at the human-animal-ecosystem interface [41]. Our results suggest that paying attention to potential threat of emerging/re-emerging pathogens in the blood, avoiding blood transfusions for immunodeficient patients and analyzing the origin of pathogens based on zoonosis may further ensure the safety of blood transfusions.

Besides, we found that the abundance of B19V was higher than others by the investigation of the microbiome. The infection of B19V can occur all over the world [24]. It is worth noting that B19V can be transmitted through transfusions of blood and blood products. Currently, B19V has been detected in various blood products, especially human coagulation factor VII, human coagulation factor IX, and prothrombin complex [42]. It was reported that the load of B19V detected in some blood products produced by Chinese manufacturers was higher than the threshold proposed by European and American countries [43,44]. In order to ensure the transfusion safety, Germany and Japan have included the detection of B19V in their routine blood screening programs [45], while China has not yet done so. Although some studies have found that the prevalence of B19V in Chinese voluntary blood donors is lower than that of the general population, and the viral load of infected people is generally not high [24], recipients with immune impairment still have a risk of infection. The qPCR detection method used in this study can amplify three genotypes of B19V using conserved primers, with high specificity and sensitivity. There were 9 B19V nucleic acid positive samples found to belong to B19-1A subtype through nested PCR amplification and sequencing, indicating that the most prevalent of B19V in China was genotype 1A. In addition, the overall prevalence of B19V was relatively low (0.07%) and varied among different regions, with the highest prevalence rate of 0.5% in Dehong Prefecture, requiring meaningful follow-up and investigation research. In short, in order to ensure blood safety and recipient safety, it is recommended to screen for B19V in voluntary blood donors.

In conclusion, our study found that emerging/re-emerging pathogens were widely present in plasma samples from healthy voluntary blood donors in seven regions of China. Some pathogens might pose potential risks to blood safety, such as *Serratia spp., Pseudomonas spp., Burkholderia spp., Leishmania spp, T. gondii* and B19V. What is more, it was found that the abundance of certain pathogens had significant regional differences, such as B19V, the proportion of which in Dehong Prefecture was much higher than in other regions. *Leishmania spp.* accounted for a high proportion of the microbes in Mudanjiang. The proportion of *S. dysgalactiae* in Mianyang was relatively high, while the one in other regions was <0.01%. The reasons for regional differences in pathogen abundance in China may be natural endemism or statistical endemism. The natural endemism refers to the distribution of some diseases being limited to certain regions due to natural conditions and statistical endemism means that the incidence rate of some diseases in some regions is significantly higher than that in other regions for a long time due to different social factors such as lifestyle, sanitary conditions or religious beliefs. According to our results, it is suggested that we should pay attention to emerging/re-emerging pathogens in different regions and certain emerging/re-emerging pathogens should be included in the blood screening protocols in corresponding regions.

However, there are some limitations in this study. Firstly, due to the ease of RNA degradation, this study only extracted DNA from blood samples, resulting in the exclusion of transfusion related RNA viruses such as DENV and WNV. Secondly, the pathogenicity of the pathogens found in this study has not been verified, and further follow-up investigation is needed. In subsequent investigations, it is great important to conduct more comprehensive national blood metagenomic testing to identify emerging/re-emerging pathogens and assess their risks in different regions. In addition, we will expand our research scope to identify zoonotic pathogens in the blood of donors' pets, so as to determine the origin of some microbes discovered in voluntary blood donors.

In summary, this study can provide a reference for the monitoring of certain specific pathogens and data support for establishing a comprehensive B19V detection system.

The following are the supplementary data related to this article.

Supplementary Table 1 The Genbank accession numbers for B19V reference sequence. Supplementary Table 1

Supplementary Table 2 High through-put sequencing results of blood samples from blood donors in seven regions of China Supplementary Table 2

Supplementary Fig. 1 The sequences of microbiome data in blood samples collected by blood donors from seven regions in China Supplementary Fig. 1

**Supplementary Fig. 2:**
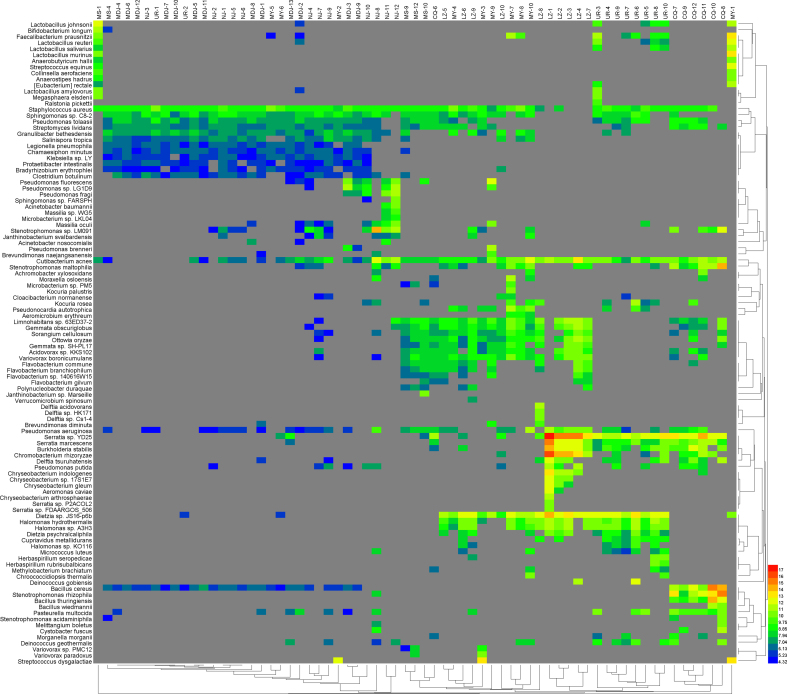
Heat map of major bacterial abundances

**Supplementary Fig. 3:**
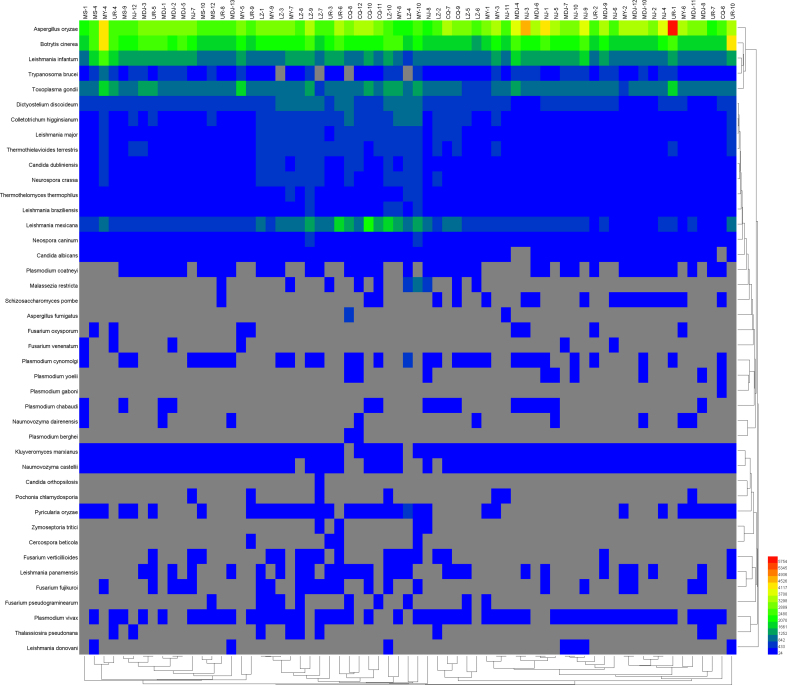
Heat map of major fungi and parasites abundances

**Supplementary Fig. 4:**
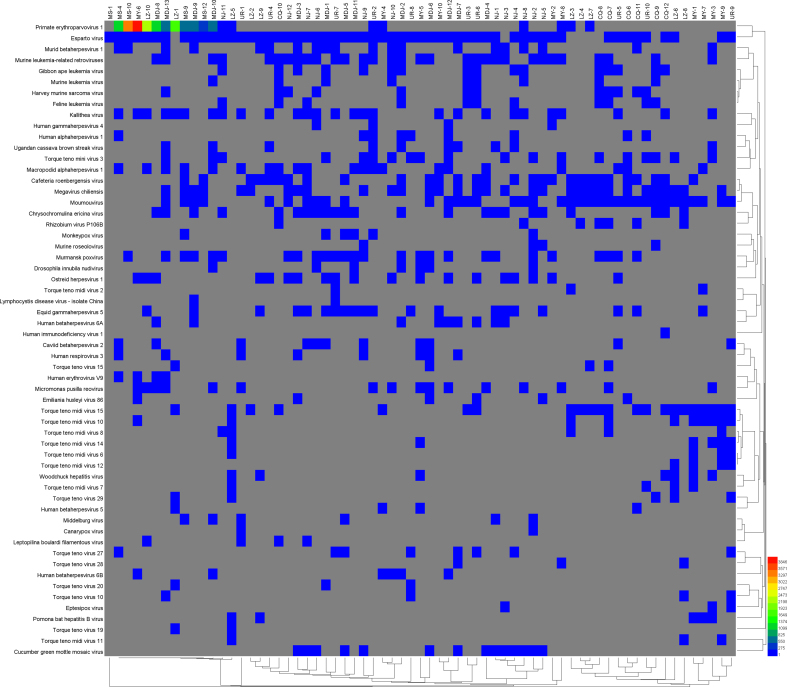
Heat map of major virus abundances

**Supplementary Fig. 5:**
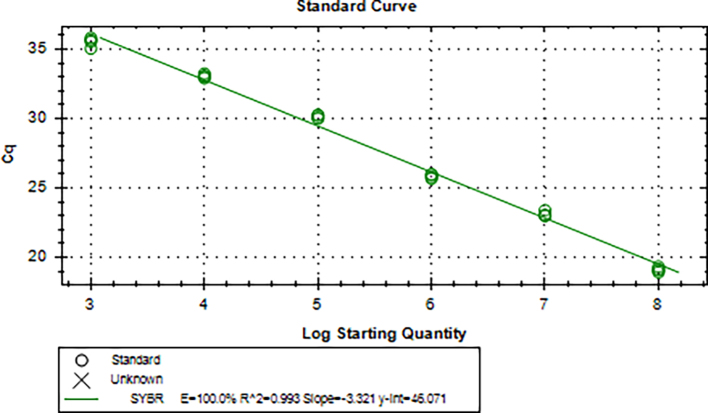
Standard curve of B19V detected by qPCR

**Supplementary Fig. 6:**
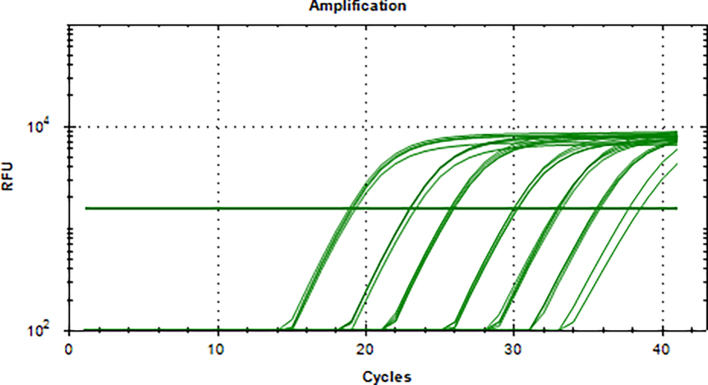
Detection of DNA specific amplification curves of different standards by qPCR

**Supplementary Fig. 7:**
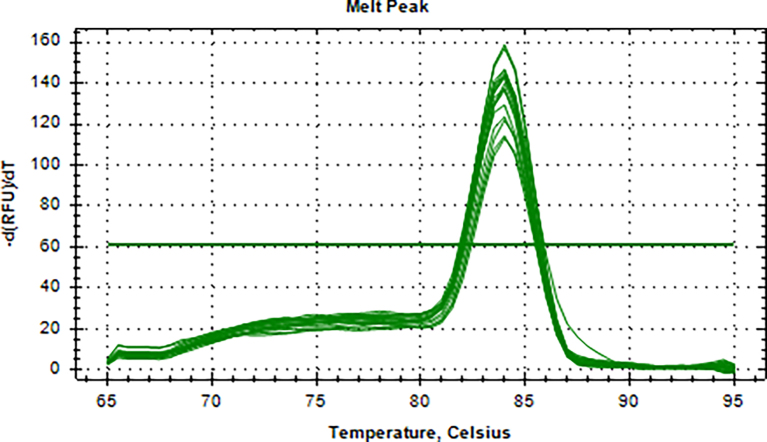
DNA specific melting curves of different standards were detected by qPCR

**Supplementary Fig. 8:**
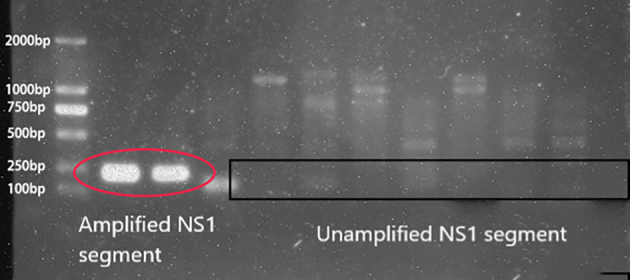
Agarose gel electrophoresis analysis of B19V genome fragment of B19V positive samples

**Supplementary Fig. 9:**
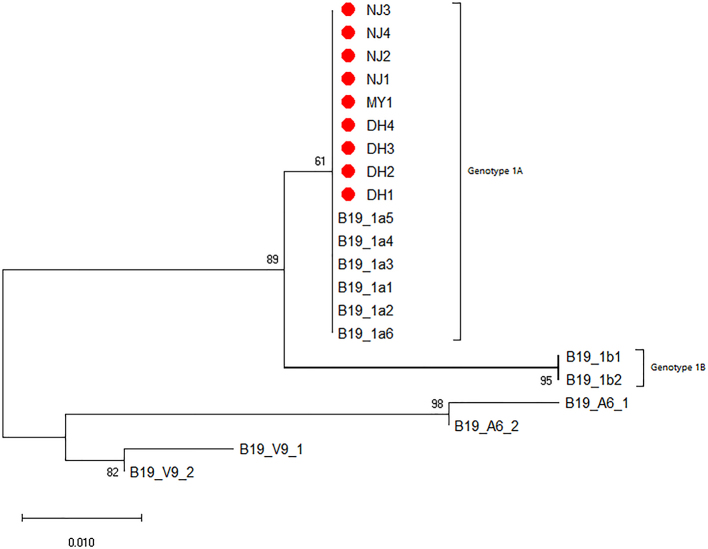
Figure Demographic characteristics of 9 B19V nucleic acid positive blood donors

Supplementary Fig. 10 Phylogenetic relationships of NS1 region of different clones Supplementary Fig. 10

## Funding

This work was supported by the CAMS Initiative for Innovative Medicine (CAMS-2021-I2M-1-060), the Key Research and Development Projects of Science and Technology Department of Sichuan Province (Grant No.2019YFS0319), and National Key Research and Development Program of China (Grant No. 2018YFE0107500).

## Author statement

Zhao Mengyi: Writing-original draft and Investigation.

LI Yuhui: Investigation and Data curation.

GAO Zhan: Data curation and Methodology.

LIU Anqing:Writing-review & editing.

LI Yujia: Validation.

LI Shilin: Resources.

Gao Lei: Investigation.

Yue Lan: Software and Formal analysis.

HUANG Mei: Resources.

WAN Jianhua: Resources.

HE Weilan: Resources.

MAO Wei: Resources.

CAI Jie: Resources.

ZHOU Jingyu: Resources.

YIN Yijing: Resources.

GUO Yanli: Resources.

ZHONG Qiulei: Investigation.

HUANG Yang: Data curation.

CHEN Limin: Writing-review & editing.

FAN Zhenxin: Conceptualization and Writing-review & editing.

HE Miao: Conceptualization, Writing-review & editing, Funding acquisition and Project administration.

## Declaration of Competing Interest

The authors report there are no competing interests to declare.

## Data Availability

The datasets presented in this study can be found in online repositories. The names of the repository/repositories and accession number(s) can be found below: https://bigd.big.ac.cn/gsa/browse/CRA006191 and the assigned accession of the submission is: CRA006191.
