# Supplementary material for: Plasma metagenomics reveals regional variations of emerging and re-emerging pathogens in Chinese blood donors with an emphasis on human parvovirus B19
Source: One Health. 2023 Jul 13;17:100602. doi: 10.1016/j.onehlt.2023.100602 (PMC10372899; doi:10.1016/j.onehlt.2023.100602)
Supplement: Supplementary Table 1 — The Genbank accession numbers for B19V reference sequence. [file mmc1.docx]

| Sequence naming | Accession NO. |
| --- | --- |
| B19_1a1 | AB030694 |
| B19_1a2 | AF113323 |
| B19_1a3 | M13178 |
| B19_1a4 | DQ225148 |
| B19_1a5 | DQ225149 |
| B19_1a6 | DQ225150 |
| B19_1b1 | DQ357064 |
| B19_1b2 | DQ357065 |
| B19_A6_1 | AY064476 |
| B19_A6_2 | AY044266 |
| B19_V9_1 | AJ249437 |
| B19_V9_2 | AY083234 |

S1 Table The Genbank accession numbers for B19V reference sequence
