# Supplementary material for: Plasma metagenomics reveals regional variations of emerging and re-emerging pathogens in Chinese blood donors with an emphasis on human parvovirus B19
Source: One Health. 2023 Jul 13;17:100602. doi: 10.1016/j.onehlt.2023.100602 (PMC10372899; doi:10.1016/j.onehlt.2023.100602)
Supplement: Supplementary Table 2 — High through-put sequencing results of blood samples from blood donors in seven regions of China [file mmc2.docx]

S2 Table High through-put sequencing results of blood samples from blood donors in seven regions of China

| Library type | Raw Reads | Clean Reads | Raw Base* | Clean Base* |
| --- | --- | --- | --- | --- |
| Metagenomic library | 6,450,647,493 | 3,446,309,917 | 1935.2 | 1033.87 |

* Raw Base and Clean Base represent the total bases of the sequencing specified in G .
