# Supplementary material for: Plasma metagenomics reveals regional variations of emerging and re-emerging pathogens in Chinese blood donors with an emphasis on human parvovirus B19
Source: One Health. 2023 Jul 13;17:100602. doi: 10.1016/j.onehlt.2023.100602 (PMC10372899; doi:10.1016/j.onehlt.2023.100602)
Supplement: Supplementary Fig. 10 — Phylogenetic relationships of NS1 region of different clones [file mmc12.docx]

S8 Figure: Demographic characteristics of 9 B19V nucleic acid positive blood donors
